# The Petrified Self 10 Years After: Current Evidence for *Mnemonic anosognosia*

**DOI:** 10.3389/fpsyg.2020.00465

**Published:** 2020-03-17

**Authors:** Sabrina Lenzoni, Robin G. Morris, Daniel C. Mograbi

**Affiliations:** ^1^Department of Psychology, Pontifícia Universidade Católica, Rio de Janeiro, Brazil; ^2^Department of Psychology, Institute of Psychiatry, Psychology and Neuroscience, King’s College London, London, United Kingdom

**Keywords:** anosognosia, awareness, memory, Alzheimer’s disease, dementia

## Abstract

Lack of awareness about disease, its symptoms and consequences, also termed anosognosia, is a common feature of Alzheimer’s disease (AD). It has been hypothesized that memory disorder may be a key contributing factor to anosognosia, with people with AD not being able to update their personal information about performance and relying on older consolidated material about ability. This potentially outdated sense of self has been named, as a metaphor, the petrified self. In the current review, evidence from the past 10 years in relation to this concept is critically appraised. In particular, focus is given to empirical evidence produced on anterograde memory deficits about performance, the profile of autobiographical retrograde memory loss and the role of frontal lobes in anosognosia in AD. Finally, wider consequences of this metaphor for the understanding of selfhood in dementia are discussed.

## Introduction

Whilst anosognosia is more generally defined as lack of awareness about neurological impairment or illness, it also can be applied specifically to Alzheimer’s disease (AD), in which patients are frequently unaware of their cognitive deficits and the consequences of their clinical condition (Mograbi et al., 2009, 2012; Mograbi and Morris, 2018). This has been shown to be associated with earlier institutionalization (Horning et al., 2014) worse prognosis (Orfei et al., 2007), reduced treatment compliance (Patel and Prince, 2001), and higher exposure to dangerous behaviors (Starkstein et al., 2007). In addition, loss of awareness has been linked to greater burden in relatives or caregivers (Seltzer et al., 1997; Verhülsdonk et al., 2013).

The manner in which the neurocognitive mechanisms supporting awareness are damaged in AD has been elucidated by experimental studies and theoretical formulations. Our formulation has been the Cognitive Awareness Model (CAM; Agnew and Morris, 1998; Morris and Hannesdottir, 2004; Morris and Mograbi, 2013), where lack of explicit awareness is thought to be the result of cognitive impairments at different levels, with anosognosia being characterized by its heterogeneity. This includes different types of anosognosia, including: (1) *Primary anosognosia*, where there is either a breakdown in connectivity, leading to impairments in bottom-up integration, or top-down modulation; (2) *Executive anosognosia*, which involves dysfunction of higher-level monitoring abilities that lead to impaired self appraisal and performance evaluation; and (3) *Mnemonic anosognosia*, in which lack of awareness is caused by specific types of memory impairment.

Under this framework, *M. anosognosia* is thought to be the main type in AD and is characterized by a failure in updating and integrating personal information to a personal data base (PDB), resulting in an outdated self-concept. Here, the PDB refers to a repository of information about the self, contrasting with more general semantic memories. In a paper in which we developed this notion with reference to the supporting experimental studies, this phenomenon was given a metaphorical term, namely, *“the Petrified Self*,” (Mograbi et al., 2009). Two elements were highlighted in this “stone” metaphor: (1) Limited updates in self-concept because of anterograde amnesia caused by degeneration of neuronal structures that support declarative memory acquisition, such as the hippocampus; and (2) a preserved core of identity based on remote autobiographical memory (ABM), in particular of the semantic type, which has long been consolidated.

It is now 10 years since the Petrified Self term was used and since then a considerable amount of new evidence has been produced about the relationship between memory, self and awareness in AD. Accordingly, in the current review, we consider this evidence, including data about ABM, as well as new insights into the relationship between anterograde amnesia and anosognosia in AD. In addition, as a comparison, the mounting neuroimaging evidence about the role of the frontal lobes in anosognosia in AD is appraised. We conclude by discussing reactions to the Petrified Self metaphor and potential implications for how we view people with dementia.

## The Remote Self in AD

A key notion of the Petrified Self is that personal knowledge is shaped by or even represented in the form of remote memories. In the last decade, several studies have tried to elucidate the temporal pattern of ABM deficits across different lifetime periods in AD. Specifically, retrieval of remote material seems to be better preserved than recent memories. Overall, it has been highlighted that in AD ABM impairments are characterized by a temporal gradient for both episodic and semantic components (Leyhe et al., 2009; Thomann et al., 2012; De Simone et al., 2016; Kirk and Berntsen, 2018).

Only few studies reported no differences in memory retrieval across different lifetime epochs (e.g., Irish et al., 2011a), despite a poorer performance in comparison to healthy controls (Irish et al., 2014, 2018). It is possible that this is due to methodological reasons. For instance, it has been previously shown that the use of the Autobiophical Interview (AI; Levine et al., 2002) tends to diminish the temporal gradient due to fewer memories being allocated to a higher number of epochs (Barnabe et al., 2012).

Furthermore, recent evidence supports multiple trace theory (MTT), according to which semantic memory retrieval is independent from the hippocampus and mediated by the neocortex after a certain consolidation period (Moscovitch et al., 2005a), while episodic retrieval is subserved by medial temporal lobe/hippocampus regardless of the lifetime period of acquisition (Moscovitch et al., 2005b). For example, the presence of a temporal gradient is more consistent for episodic memory (Philippi et al., 2012), including vividness and details specificity (Donix et al., 2010; Irish et al., 2011b; Seidl et al., 2011; Müller et al., 2016), while personal semantics can be relatively preserved in AD (Martinelli et al., 2013). Moreover, it has been suggested that the degree of impairment of semantic ABM may depend on the stage of the disease. While episodic autobiographical memory can be affected in early AD, semantic components may be preserved, becoming impaired only in more severe stages of the condition (Seidl et al., 2011; Kirk and Berntsen, 2018). The temporal gradient is also evident from the early stages of dementia for episodic ABM, while semantic ABM seems to be characterized by a flatter distribution across lifetime periods (Seidl et al., 2011). Therefore, a decline in semantic ABM appears to be dissociated from damage to the primary episodic memory support structures, such as the hippocampus, being affected by later neocortex degeneration.

Although longitudinal studies of autobiographical memory in AD are scarce (e.g., Starkstein et al., 2005), cross-sectional comparisons between AD and MCI may illuminate the change in the content of memories over time with the progression of the condition. The general findings indicate that episodic memory is impaired across life epochs in AD in relation to MCI, but that remote semantic memories are preserved at similar levels in comparison to MCI (Leyhe et al., 2009; Hirjak et al., 2017) and even healthy controls (Thomann et al., 2012). This suggests that autobiographical memory deteriorates as a function of dementia severity, but that episodic impairments are seen from the earlier stages of the condition, whereas autobiographical semantic loss is observed only later on in the course of the illness.

Interestingly, it has been reported that remote memories are proportionally more frequently retrieved by people with AD (De Simone et al., 2016; Müller et al., 2016) and that detail specificity is positively associated to retrieval frequency of memories (Müller et al., 2016). Hence, retrieval frequency may modulate vividness and temporal gradient effects, as a result of the semanticisation process of more frequently retrieved memories, which would gradually acquire independence from medial temporal structures.

This notion is also supported by evidence from an fMRI investigation of the neural correlates of ABM in AD (Meulenbroek et al., 2010). The study showed enhanced activation of frontal regions (inferior frontal gyrus, ventromedial prefrontal cortex) which was inversely associated with hippocampal volume, suggesting that following hippocampal degeneration memory retrieval may rely more on frontal structures, mediating the activation of more preserved memories that probably have undergone semanticisation (Meulenbroek et al., 2010). Moreover, it has been shown that episodic, but not semantic, ABM retrieval impairments are associated with changes in hippocampal morphology (Thomann et al., 2012).

Recent research on the extent to which the hippocampus is involved in ABM revealed opposite results. Philippi et al. (2012) reported that the left hippocampus is associated to remote memory retrieval, while the right hippocampus is correlated with retrieval of more recent memories. Additionally, the authors hypothesize the presence of a rostrocaudal gradient depending on retention duration: lesions to left anterior regions are implicated in impairments of remote memories retrieval while more posterior lesions are linked to deficits in encoding, consolidation or retrieval of recent memories. Another study reported that remote episodic memory retrieval correlates with lateral and left posterior hippocampus (including CA1-3 and subiculum), while more recent memories relied on the left hippocampal head (border of CA1, CA2, and subiculum), in a sample composed by healthy older adults, MCI, and AD (Thomann et al., 2012).

Taken together, these findings describe the profile of ABM impairment in AD, which may play a pivotal role in self-knowledge and self-continuity in this condition. In fact, recent evidence supports a bidirectional relation between the self and memory. Martinelli et al. (2013) showed that AD is characterized by the lack of relation between autobiographical episodes, self-concept and self-defining memories typically seen in healthy individuals. Moreoever, AD patients show alterations in strength and complexity of their sense of self and tend to produce fewer memories tied to the self, with self-concept changes being associated with lower memory integration abilities (Ben Malek et al., 2019). Alterations of the self in AD have also been shown to be associated to memories characterized by lower specificity, fewer contextual details (El Haj and Antoine, 2017) and self-defining memory episodicity (Martinelli et al., 2013). Crucially, memory deficits in AD patients appear to interfere with the ability to remember and acknowledge how past events define themselves.

It is important to highlight that these changes are relative, typically defined in comparison to healthy older adults. Research into life stories (El Haj et al., 2019) has shown AD patients with mild dementia can retrieve ABMs to reflect on self-continuity, being able to maintain a life story and, interestingly, to be more concerned about changes in self-continuity as compared as healthy controls. This suggests that self-concept in AD, particularly during the milder stages of the condition, does not remain unchanged, a common misinterpretation of the Petrified self metaphor. These changes, however, are mediated by the profile of memory impairment of AD.

Finally it is worth noting that research on ABM has also been approached in its relation with future episodic simulation, suggesting a similarity between remembering the past and imaging the future in AD (Addis et al., 2009; El Haj et al., 2015a, b). Specifically, El Haj et al. (2015b) showed that AD patients tend to evoke similar themes during past and future thinking, with this pattern also extending to self-defining memories. In the case of AD, this may contribute to a lack of appreciation of future consequences of their condition, projecting self-concepts tied to remote memories into future imaging.

## Anterograde Amnesia and Anosognosia in AD

Evidence from the past 10 years strongly supports the notion that, despite showing fairly accurate predictions of performance, people with AD exhibit a failure in transferring information from online performance and actual experience to the PDB, resulting in a stable but outdated self-evaluation. The metamemory literature shows that AD patients can make accurate predictions about their performance (Gallo et al., 2012; Bertrand et al., 2018; Bertrand J. M. et al., 2019) and use appropriately extrinsic and intrinsic factors in these predictions (Thomas et al., 2013; Rosen et al., 2014). Moreover, it has been shown that metacognitive judgments in AD are similar regardless the presence of feedback (Cosentino et al., 2016; Chapman et al., 2018), thus suggesting a failure of integrating information about ongoing performance to make more accurate predictions. Crucially, it has been shown that even when prediction accuracy is higher in post- than in pre-test conditions, after 1 h delay AD patients estimation return to be as low as in the pre-test condition (Stewart et al., 2010). This suggests that performance monitoring is fairly preserved in AD and that metacognitive impairments in this group may derive from lack of updating of personal information.

Dodson et al. (2011) reported impairments in episodic memory monitoring in AD for item-by-item confidence accuracy, but accurate predictions at task level. They also observe that in a condition of additional exposure to test material, there is improved memory performance in AD, that does not differ from normal controls, but that still is accompanied by metamemory deficits. Similarly, in a study investigating metacognitive abilities through an associative learning paradigm, AD patients showed reduced online monitoring, presenting impairments in feeling of knowing and retrospective judgments in item-by-item judgments, but preserved sensitivity to extrinsic and intrinsic factors and feedback when asked to predict general performance (Rosen et al., 2014). Despite important methodological issues affecting results, such as procedure complexity and task difficulty, findings from Dodson et al. (2011) and Rosen et al. (2014) suggest that although patients may have monitoring impairments, there is still some preserved calibration, with patients being able to revise their initial estimations of ability, particularly when prompted about performance.

Further insight comes from research investigating neuropsychological intervention outcomes for memory and metamemory abilities (Silva et al., 2017). The authors compared judgments of learning about memory performance before and after cognitive training. Pre-test scores showed that AD patients tend to overestimate their memory performance. Post-training scores revealed that the training improve both memory and metamemory scores, but prediction of performance continued to be an overestimation of actual abilities. People with AD are able to retain online metamemory information, but this is not incorporated into longer term representations.

## The Role of the Frontal Lobes Revisited

One aspect not fully developed in the 2009 article referred to the role of the frontal lobes in anosognosia in relation to AD. In that context, three main hypotheses were suggested for an association between frontal lobe dysfunction and AD anosognosia: difficulties in error monitoring, impairments in memory retrieval and alterations in belief evaluation systems. Emerging new evidence, mainly from neuroimaging studies, allows a critical revision of these notions.

Structural imaging studies investigating the relation between gray matter volume changes and anosognosia in AD mainly reported an association between frontal atrophy and self-awareness. In particular, anterior cingulate cortex integrity has been associated to lack of awareness (Guerrier et al., 2018) and metamemory deficits (Bertrand et al., 2018). Another study analyzing metamemory abilities reported an association with right insula volume but also strong correlations between anterior and posterior cingulate cortex that may have been significant with a larger sample size (Cosentino et al., 2015). Interestingly, Spalletta et al. (2014) investigated anosognosia-related structural changes in amnestic mild cognitive impairment (MCI) patients, comparing patients who converted (CONV) to AD and those who did not (NON-CONV) after 5 years. Their results show different relations with anosognosia for the two groups: specifically, awareness for the memory domain in the CONV group was associated with anterior cingulate cortex (ACC) and inferior frontal gyrus volume, while temporal structures were associated to different awareness measures in the NON-CONV group.

Further evidence for frontal involvement comes from research on dementia subtypes, including AD, frontotemporal dementia and primary progressive aphasia (Shany-Ur et al., 2014), indicating that the tendency to overestimate overall functioning is associated to changes in cortical and subcortical frontal regions. Only one study reported divergent results, suggesting that anosognosia in AD and MCI is mediated by temporal degeneration, including the hippocampus (Tondelli et al., 2018). Surprisingly, Senturk et al. (2017) found no correlation between cortical thickness and anosognosia in early AD and amnestic MCI patients, possibly due to sample characteristics, such as dementia severity, methodological issues (e.g., ROIs choice) and the potential contribution of non-cognitive factors to anosognosia.

Task-related functional magnetic resonance imaging (fMRI) studies highlight the role of the frontal lobes, in particular ventromedial prefrontal cortex (VMPFC) and ACC, for self-referential processes in relation to unawareness in AD (Zamboni et al., 2013; Genon et al., 2014). Moreover, Amanzio et al. (2011) found differences in functional activation during a response inhibition task between AD patients with preserved and impaired awareness, indicating reduced recruitment of frontocingulate, parietal and temporal areas for the unaware AD group. Positron-emission tomography (PET) studies consistently reported that frontal lobe dysfunction has been associated with anosognosia, with hypometabolism in dorsal ACC (Guerrier et al., 2018), dorsomedial PFC and superior frontal sulcus (Jedidi et al., 2014), orbitofrontal cortex and posterior regions as posterior cingulate cortex (PCC) and precuneus (Perrotin et al., 2015). Interestingly, evidence from single-photon emission computed tomography (SPECT) research investigating regional cerebral blood flow (rCBF) suggests that anosognosia in AD is not only related to frontal dysfunction but also to compensational mechanisms reflected by higher rCBF in parieto-occipital regions (Tagai et al., 2018). However, further research is needed to confirm this hypothesis.

Lastly, resting-state functional connectivity maps studies provide crucial evidence elucidating the neural correlates of anosognosia in AD, suggesting that unawareness may emerge from decreased interregional connectivity between and within medial prefrontal cortex and medial temporal regions. In particular, it has been shown a relation between memory self-appraisal and decreased MPFC connectivity with dorsolateral PFC, anterior cingulate cortex and hippocampus (Ries et al., 2012) and an association between anosognosia and disrupted connectivity between PCC and orbitofrontal cortex (OFC) and between OFC and hippocampus (Perrotin et al., 2015). A study conducted by Berlingeri et al. (2015) found significant functional connectivity reduction in unaware AD patients within the inferior medial temporal cortex (IMTC) and VMPFC networks, but only disconnection between IMTC and hippocampus and insular cortex correlated with anosognosia severity. Finally, a more recent study reported that memory awareness correlated with the degree of disconnection between hippocampus and retrosplenial cortex extending to the ventral PCC and right posterior inferior parietal lobe; in addition, anosognosia was associated with decreased connectivity between hippocampus and VMPFC (Antoine et al., 2019).

Taken together, the evidence suggests that default mode networks (DMN) alterations may mediate self-appraisal and self-knowledge about cognitive functioning in dementia. This may reflect the pivotal role of frontal dysfunction in impaired self-awareness, with frontal lobe alterations leading to executive anosognosia (Morris and Mograbi, 2013; Mograbi and Morris, 2014). Moreover, the fMRI evidence reviewed is in line with the current notion that executive functions are mediated by anterior-posterior connectivity, thus indicating that anosognosia may be linked to disconnection within a monitoring network. In addition, the association between self-awareness deficits with medial temporal atrophy and intraregional connectivity suggests that anosognosia in AD may also depend on mnemonic dysfunction. The involvement of memory impairment in anosognosia in AD is further reinforced by evidence of decreased connectivity between frontal and temporal regions, indicating that a disconnection process can lead to alterations of self-appraisal and self-awareness. This may reflect, for instance, limited access to incident memory when engaging and self-evaluation processes. Conversely, lack of input from monitoring processes may contribute to failures in updating knowledge about the self. In any case, the possibility of unawareness emerging as a disconnection syndrome should be further explored in studies using structural and functional neuroimaging approaches.

The role of the frontal lobes in beliefs evaluation systems remains little explored in relation to anosognosia in AD. This has been suggested as an important factor in cases of unawareness for other clinical conditions, such as in stroke (Vuilleumier, 2004), with some empirical evidence supporting the notion (e.g., Venneri and Shanks, 2004; Vocat et al., 2013). It has been suggested that global measures of awareness in AD may be more vulnerable to beliefs (Chapman et al., 2018), but future studies are needed to investigate this issue.

**FIGURE 1:**
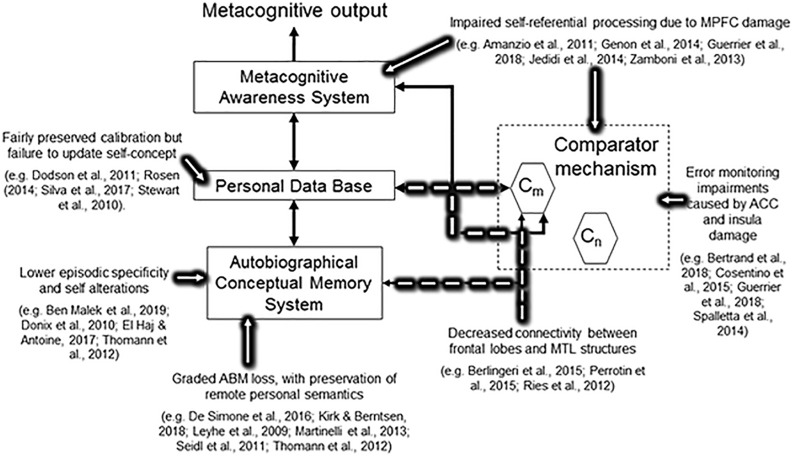
Simplified version of the Cognitive Awareness Model showing current evidence in relation to the main concepts of the Petrified Self.

Figure 1 summarizes the reviewed evidence in relation to the Petrified self concept.

## What Is in a Metaphor and Concluding Thoughts

The notion of a “petrified self” was first suggested as a metaphor for a self-concept that was stable, but outdated in time. Metaphors are literally false. The idea was highlighting a core identity in people with AD, based on autobiographical memory material that had been long consolidated, while, at the same time, acknowledging the difficulties caused by anterograde amnesia in the updating of self-concept. These difficulties have been suggested not only in the case of AD (e.g., Leyhe et al., 2009; Thomann et al., 2012; De Simone et al., 2016; Kirk and Berntsen, 2018), but also in other cases of hippocampal amnesia (e.g., Rosenbaum et al., 2005, but see also Elward and Vargha-Khadem, 2018).

What was never implied in the metaphor was that people with AD are dead inside, ossified or immune to change. There is, in fact, important evidence that contradicts these notions. For example, all rehabilitation and cognitive stimulation efforts (e.g., Bertrand E. et al., 2019) attest to the potential of improvement of people with dementia and other patients with amnesia (Wilson, 2009). Specifically related to awareness of difficulties, the notion of implicit awareness (Morris and Mograbi, 2013) suggests that behavioral and affective change may happen without explicit knowledge by patients. Similarly, emergent awareness (Moro, 2013) indicates that engaging in activities linked to deficits may promote increased awareness.

Additionally, it is important to highlight that the metaphor applies to narrative aspects of the self, i.e., those that are more directly linked to verbal questioning of self-concept. Selfhood is a complex phenomenon, with many different “selves” or self processes, as indicated in the original formulation about the petrified self (Mograbi et al., 2009). Certain aspects of the self, such as bodily awareness, may remain preserved even in the final stages of AD. Our theoretical and methodological perspective also makes us privilege the impact of neurobiological factors on the self, but selfhood also emerges from other sources, such as social interaction (e.g., interpersonal self; Baird and Thompson, 2018).

The field of dementia studies has demonstrated the importance of terminology and its potential impact on malignant social psychology. While care should be taken in how we use words to describe the condition, it is also important not to deny the profile of cognitive impairments that may affect selfhood in dementia. When allied with a careful approach, awareness can be a powerful tool for understanding or improvement.

## Author Contributions

SL, RM, and DM participated in writing up and revising the manuscript.

## Conflict of Interest

The authors declare that the research was conducted in the absence of any commercial or financial relationships that could be construed as a potential conflict of interest.
